# Image-guided video-assisted thoracoscopic resection (iVATS): Translation to clinical practice—real-world experience

**DOI:** 10.1002/jso.25897

**Published:** 2020-03-12

**Authors:** Ritu R. Gill, Julianne Barlow, Michael T. Jaklitsch, Eric J. Schmidlin, Phillip M. Hartigan, Raphael Bueno

**Affiliations:** 1 Department of Radiology, Beth Israel Deaconess Medical Center, Boston, Massachusetts; 2 Department of Surgery, Brigham & Women’s Hospital, Boston, Massachusetts; 3 Department of Radiology, Brigham & Women’s Hospital, Boston, Massachusetts; 4 Department of Anesthesiology Perioperative and Pain Medicine, Brigham & Women’s Hospital, Boston, Massachusetts

**Keywords:** advanced image-guided operating room, C-arm CT, fiducials, hybrid operating room, lung cancer, VATS

## Abstract

**Objective:**

We developed a novel approach for localization and resection of lung nodules, using image-guided video-assisted thoracoscopic surgery (iVATS). We report our experience of translating iVATS into clinical care.

**Methods:**

Methodology and workflow for iVATS developed as part of the Phase I/II trial were used to train surgeons, radiologists, anesthesiologists, and radiology technologists. Radiation dose, time from induction to incision, placement of T-bar to incision and incision to closure, hospital stay, and complication rates were recorded.

**Results:**

Fifty patients underwent iVATS for resection of 54 nodules in a clinical hybrid operating room (OR) by six surgeons. Fifty-two (97%) nodules were successfully resected. Forty-two (84%) patients underwent wedge resection, four (7%) lobectomies, and two (4%) segmentectomy all with lymph node dissection. Median time from induction to incision was 89 minutes (range: 13–256 minutes); T-bar placement was 14 minutes (10–29 minutes); and incision to closure, 107 minutes (41–302 minutes). Average and total procedure radiation dose were: median = 6 mSieverts (range: 2.9–35 mSieverts). No deaths were reported and median length of stay was 3 days (range: 1–12 days).

**Conclusions:**

Translation of iVATS into clinical practice has been initiated using a safe step-wise process, combining intraoperative C-arm computed tomography scanning and thoracoscopic surgery in a hybrid OR.

## 1 | INTRODUCTION

Lung cancer is associated with high morbidity, mortality, and cost of care, and it is the second most common cancer by incidence in the United States.^1^ In 2020, the estimated number of new cases of lung cancer in the US alone, will be 228,820 (116300 in men and 112520 in women) and the number of deaths is projected to be 135,720 (72,500 in men and 63, 220 in women).^2^ Given the increasing costs associated with care of advanced stage lung cancer and the ability to achieve curative resection with small early stage lung cancers, there is a need for a paradigm-changing approach to help improve outcomes in lung cancer.

With the increase in annual frequency of computed tomography (CT) scan in adults over the last decade and implementation of lung cancer screening, the detection rate of lung nodules has increased from 3.9 to 6.6 per 1000 person-years.^3^ This poses a significant diagnostic burden on radiologists, pulmonologists, and surgeons. In addition, ground glass nodules have gained significant attention with the new pathological classification of lung cancer.^4,5^ Management of pulmonary nodules detected by CT depends on their size, radiographic appearance, rate of growth, and pretest probability of malignancy. Current guidelines recommend biopsy and/or surgical resection for nodules larger than 1 cm and follow-up with CT scans for smaller lesions to demonstrate stability.^6,7^ Similar criteria apply to ground glass opacities (GGO), which are less-distinct nodules that often represent carcinoma in situ or early adenocarcinoma and are usually longitudinally followed by CT scans until they grow denser and larger, and thus are more likely to be invasive cancers.^8^ Nodules that appear suspicious on imaging are often challenging from a management perspective due to small size and density, as they are difficult to biopsy successfully by image guidance and equally challenging to resect due to inability to palpate and localize using traditional surgical techniques.

There is an emerging body of research into developing and evaluating localization techniques that can help targeted resection of small lung nodules.^9–12^ These techniques are combined with surgery and include *intraoperative adjuncts* (ultrasound, fluoroscopy); *markers* placed preoperatively or intraoperatively, such as hookwires, fiducials, microcoils, or radioactive seeds; *dyes* (methylene blue and indocyanine green [ICG]); and *molecular targets* (flourophores).^13–24^ The markers (fiducials, dyes, and tracers) may be placed in the imaging suite a few hours before surgery or within the hybrid operating room (OR) just before the surgery. Many of these techniques have limitations. For example, there are concerns of spillage and leak of dye into the pleura and adjacent structures causing confusion at intraoperative localization. There have also been reported dislodgment of fiducials during transport and positioning, as well as anxiety and pain stemming from the patient waiting for surgery awake after percutaneous localization. Technically, the use of bronchoscope for localization may not be feasible in all locations within the lungs, and ultrasonography may not be effective for very small and ground glass lesions.

We developed a technique using intraoperative T-bar placement in the hybrid OR, immediately followed by surgery with the patient positioned in the surgical position.^25^ The fiducials are placed so that a suture comes out of the lung as near as possible to the nodule to help guide the surgeon as to its location and to allow the entire fiducial to be removed as part of the operation. Our next step was to adapt the technology to clinical practice and make it available for patient care. We report our experience of translating iVATS into standard clinical care.

## 2 | METHODS

### 2.1 | Training

Methodology and workflow for iVATS developed as part of the Phase I/II trial^25^ were used to train surgeons, radiologists, anesthesiologists, and radiology technologists. A detailed procedure manual was created and available for reference in the OR. A video detailing the steps of the procedure and workflow was also created and was used as educational material for both patient consent and education of radiologist, anesthesiologist, and surgeons. Three training sessions with phantoms in the advanced imaging multi-modality OR to train the technologists were also organized before enrolling patients for the current study. Additional support was provided by the team, from the initial trial with one-on-one discussion and either in person presence or availability via telephone during the procedure.

### 2.2 | Study cohort

**FIGURE 1:**
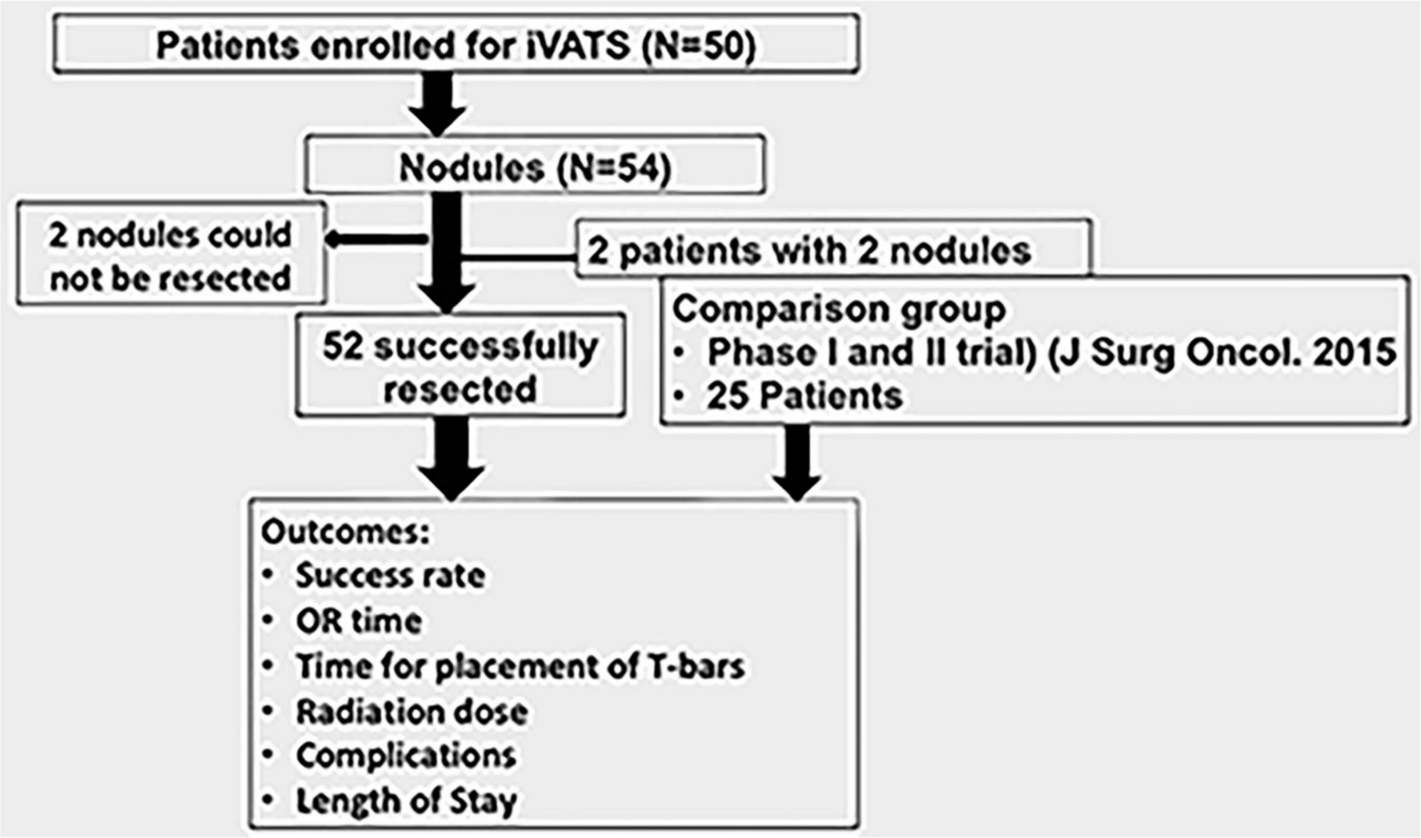
CONSORT diagram depicting the schema of the study

We prospectively offered patients with known small pulmonary nodules suspicious for malignancy, who were referred for surgery at a single academic medical center, the option to undergo iVATs. Indications for the iVATS included small nodule size, history of prior lobectomy, limited lung function, expectation for future contralateral lung resection, or patient preference. A relative contraindication was a patient with large upper body size; due to concern related to positioning for the cone beam CT. The patients underwent preadmission testing to ensure ability to undergo thoracic surgery (Figure 1).

### 2.3 | Anesthesia (positioning) and maneuvers

The patient was positioned in the lateral decubitus position in preparation for VATS resection on the operating table in the hybrid OR and placed under general anesthesia. All lines and tubes were secured and taped to allow an unhindered spin of the cone beam CT scan. The anesthesia maneuvers including breath-hold strategy were discussed with the anesthesiologist before the procedure.

After optimal positioning of the patient, the anesthesiologist ensured a full inspiratory breath-hold for the scan (5–8 seconds) by clamping the endotracheal tube at the desired tidal volume. The clamp was removed and ventilation was resumed after the scan and during the planning of the intended path for the fiducial. The anesthesiologist then clamped the lung with the nodule in the same tidal volume as the original scan, while allowing ventilation to the contralateral lung. The fiducials were then placed. The lung with the fiducials was then collapsed in preparation for the surgery.

### 2.4 | Imaging

Preoperative CT scans were used to generate three-dimensional surface models for surgical planning and for multidisciplinary discussion and planning. Patients were brought to the clinical hybrid OR suite and general anesthesia was administered, bronchoscopic examination performed, and patients were placed in the lateral decubitus position based on the side of the lesion. A C-arm CT scan of the predetermined field of view that included the nodule position was acquired. The radiologist reviewed the C-arm CT to localize the nodule and plan trajectories for percutaneous T-bar placement using iGuide needle guidance software (Siemens Healthcare AG, Forchheim, Germany). The planned needle pathways were inherently integrated into the C-arm fluoroscopic imaging system, which provides laser crosshair and guidance markers on fluoroscopy images to direct the needle pathway for T-bar placement (Kimberly-Clark, Roswell, GA).

### 2.5 | Surgery

**FIGURE 2:**
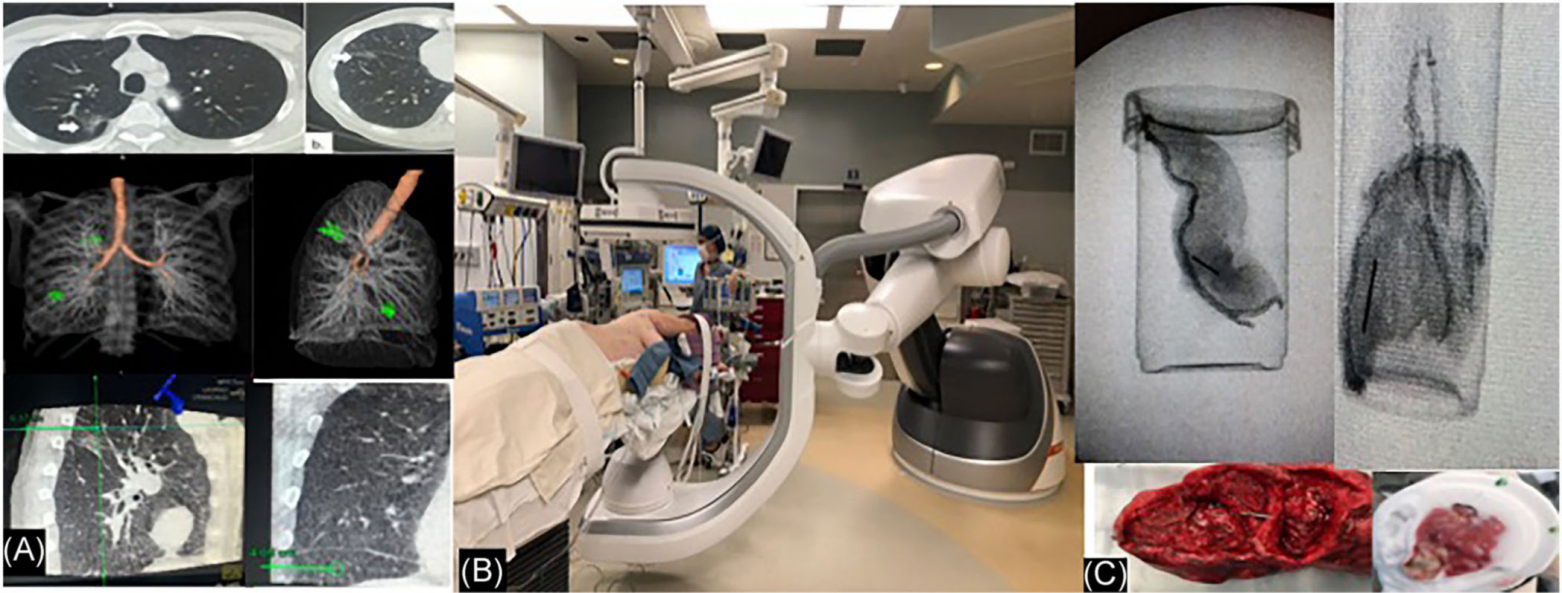
A, Axial computed tomography (CT), 3D volume rendered and sagittal and coronal cone beam CT images, of patient with two nodules resected in the same procedure; the size, morphology location, and T-bar path (green lines) are depicted

The patient was prepped and draped for VATS and a wedge resection or segmentectomy, depending on the radiographic findings, was performed with guidance and thoracoscopic visualization of the T-bar sutures. Further resection (lobectomy and segmentectomy) was based on the frozen section and intraoperative findings. Lymphadenectomy or lymph node sampling was routinely performed. The specimen and T-bars were removed using an endo-bag. A CT scan or X-ray of the excised lung resected specimen along with the T-bars was acquired in an adjoining room (away from the patient) to ensure complete excision of the T-bars and the nodule. Frozen section histological analysis provided the diagnosis and confirmed negative margins of resection. All incisions were closed, a chest tube was placed, and the patient was awakened, extubated, and transferred to the post-anesthesia care unit (Figure 2).

### 2.6 | Data collection and analysis

Radiation dose, time from induction to incision, placement of T-bar to incision, incision to closure, hospital stay, and complication rates were recorded. The total skin dose (expressed in mGy) was used as a measure of radiation exposure. Skin dose data were collected from the “‘Exam Protocol”‘ of the ARTIS Zeego and Pheeno scanners (Siemens Healthineers, Germany). Comparison was performed with outcomes of the clinical phase I and II trial (NCT01847209). Statistical analyses were using performed using JMP Pro software (version 14, Cary, NC). Descriptive statistics are reported as median (range) for continuous data and as number (%) for categorical data. The Kruskal–Wallis test was used to compare the outcomes with those from the clinical trial. The Pearson correlation coefficient was used to investigate the association between the procedure time and the surgeon’s experience. All *P*-values were two-tailed, with *P* < .05 being considered statistically significant.

## 3 | RESULTS

**FIGURE 3:**
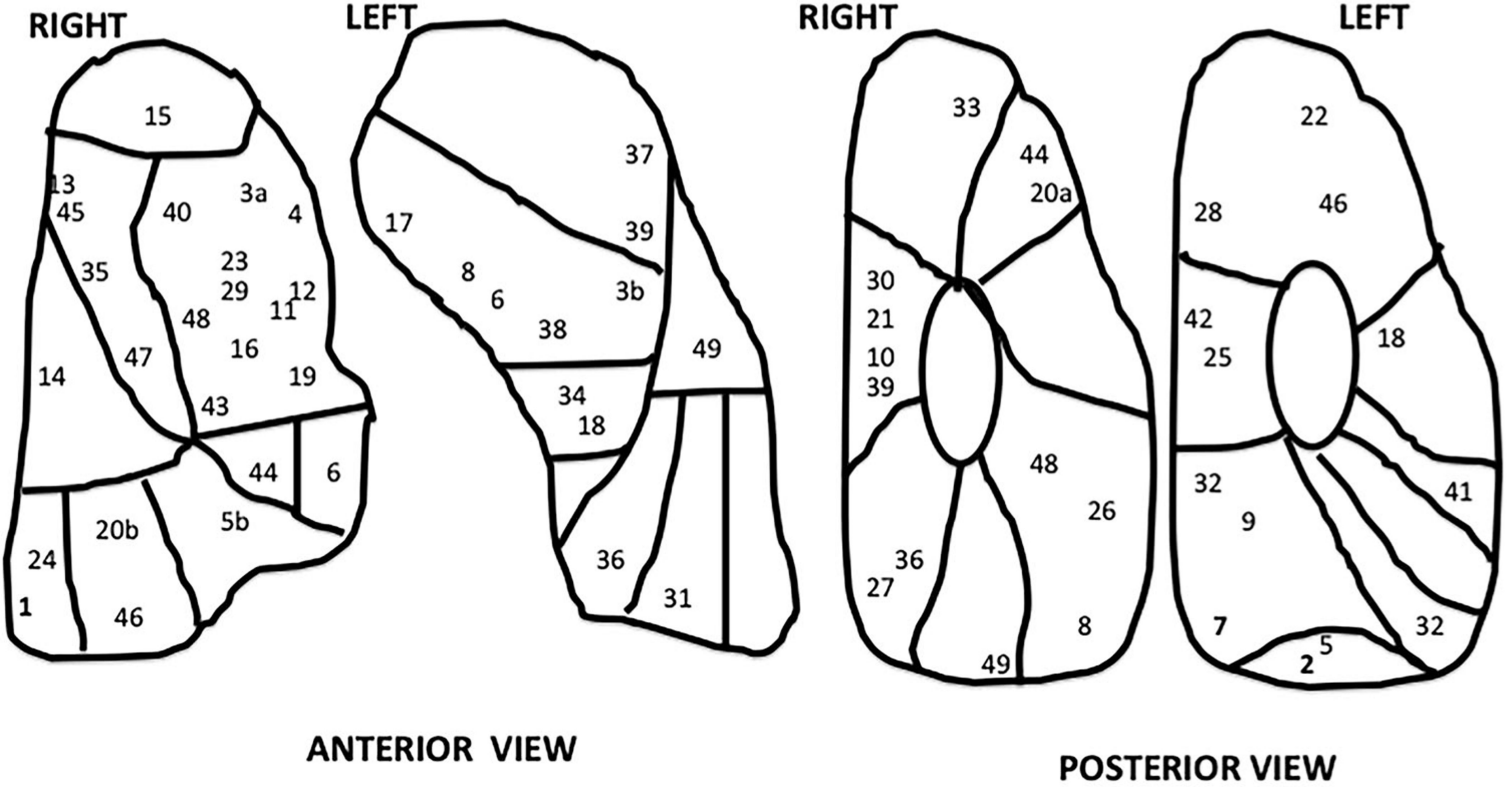
Pictorial display of the anterior and posterior surface of the lungs with all the nodule locations marked as a case number

Fifty patients underwent the iVATS procedure during the reported period and 48 patients had successful resection of their pulmonary nodules by six surgeons. Of the 50 patients who underwent surgery, 20 were men and 30 were women with a median age of 65 years (Table 1). Most patients were former cigarette smokers. By preoperative CT evaluation, 42 of the nodules were either GGOs or partially solid. The median nodule size by preoperative CT scan was 1.3 cm. Average distance of nodule from the pleura was: range = 0-4.2 cm (Figure 3).

Six thoracic surgeons, two radiologists, six anesthesiologists, and six radiology technologists were trained during the study period (2015-2019). Forty-four patients were imaged on the Artis Zeego system (Siemens Healthineers), and six patients underwent localization on the Artis Pheeno system (Siemens Healthineers). The workflow developed in the research OR was successfully translated to the clinical OR. All of the pulmonary nodules were successfully identified on intraoperative CBCT images. In 46 cases, two T-bars per nodule were placed, and in four cases, only one T-bar was needed for localization. In two cases, methylene blue was combined with T-bar foe localization and in two cases, ICG was used for both nodule localization and lymph node mapping. In four patients, two scans were performed.

Fifty-two (97%) nodules were successfully resected, one (2%) lesion could not be localized due to issues with mobility of the OR table, and one (2%) could not be resected, as the patient was unable to tolerate single lung ventilation and became unstable. In four patients, two nodules were localized with same scan (two nodules were in the separate segments within same lobe and two were in two different lobes). In one patient, the margin was positive on the final pathology, and in three patients, the second fiducial could not be placed due to pneumothorax, but localization of the nodule was feasible with one fiducial only. Eleven (20%) nodules were solid, 20 (37%) had more than 50% ground-glass component, 17 (31%) had more than 75% ground-glass component, and six (10%) nodules were 100% ground glass. Final pathology revealed that 44 (84%) were in the spectrum of adenocarcinoma, one (2%) squamous cell carcinoma, three (5%) metastases, four (8%) benign lesions, two (3%) organizing pneumonia, one (2%) pneumonia, and one (2%) lymphocytic interstitial pneumonia (Table 2).

Forty-two (84%) patients underwent wedge resection (four patients had two lesions resected by wedge resection during the same procedure), four (7%) lobectomies, and two (4%) segmentectomy. The margin for sub-lobar resections ranged between 0.8 and 4.5 cm (interquartile range: 0.9 cm). Lymph node dissection was performed in 45 (86%) patients. Four patients had significant adhesions and the localization with fiducials was found to be very helpful in successful localization of the nodules. Three T-bars could not be recovered—one was lodged in the diaphragm, one was deflected by the rib and ended in the chest wall, and the third was misplaced into the liver.

**FIGURE 4:**
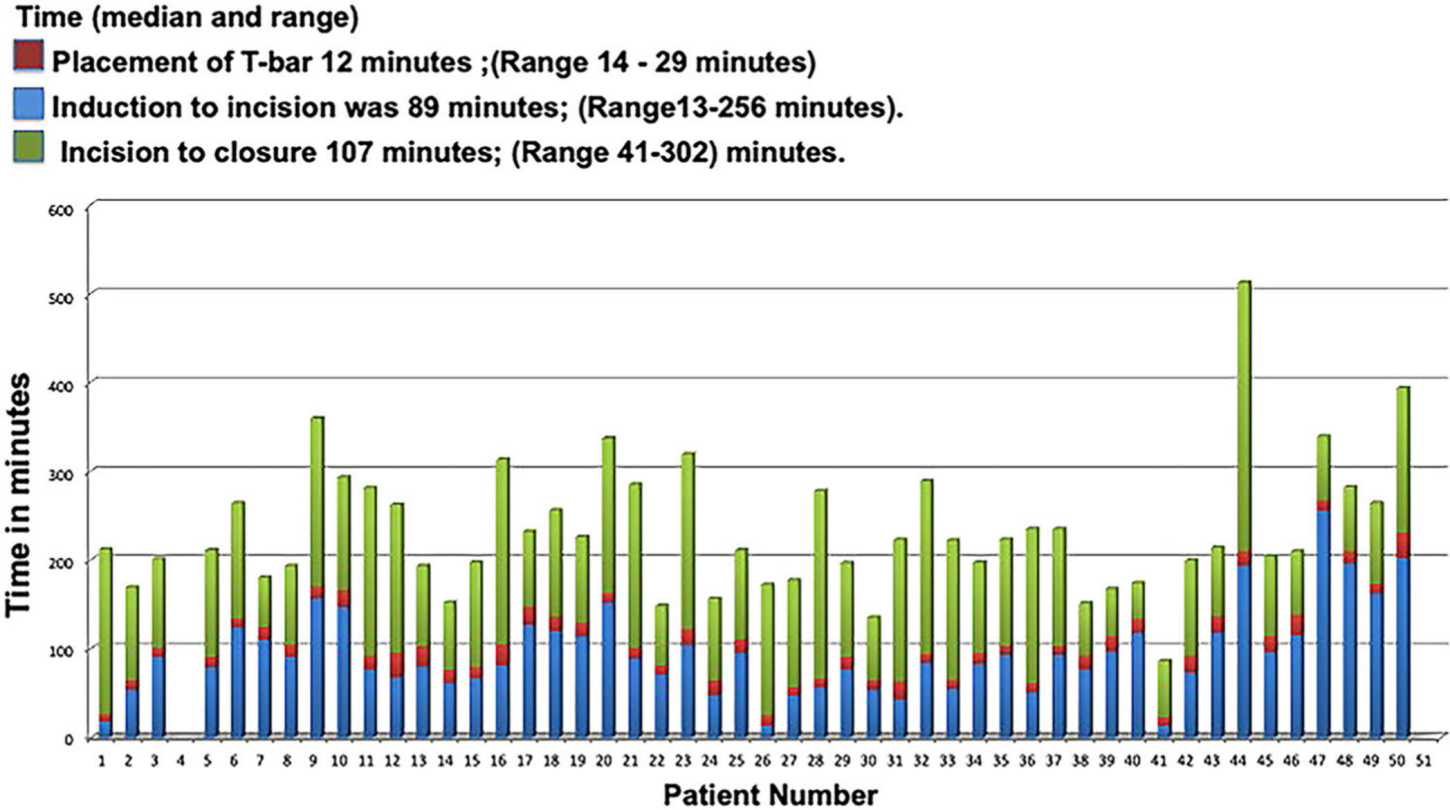
Bar graph shows the time per case with the time for each component; red color represents the time taken for T-bar placement; blue color represents the time for induction to incision; and green color represents the time for incision to closure in minutes

The median radiation dose for the procedure was 6mSieverts (range: 2.9–35mSievert). The median time from induction to incision was 89minutes (range: 13–256minutes). The median time from placement of T-bar to incision was 12 minutes (range: 4–129minutes). Median time from incision to closure was 107 minutes (range: 41–302 minutes; Figure 4). There were no perioperative deaths, and all patients were discharged from the hospital (median length of stay = 3 days, range: 1-12). There was no 30- or 90-day mortality. One patient developed pneumonia, one patient had a prolonged air leak for days, and one patient had a splenic bleed requiring embolization. Complications related to technique were retention of fiducial and positive margin in one patient and one lesion could not be successfully resected. There was no significant association between surgeon experience (number of iVATS performed) and overall surgery time (*P* = .4986).

### 3.1 | Comparison of the current study with the clinical trial (iVATS)^25^

When comparing data from the current study with those published in the original clinical trial, there was no significant difference in the induction to incision time (*P* = .522), incision to closure time (*P* = .5258), radiation exposure (*P* = .5173) and placement of T-bars (*P* = −.4714), or in induction to incision time (*P* = .3978). Average length of stay was 3 days (1-12 days). There was no significant difference between the two groups when comparing overall surgery times (*P* = .4599).

## 4 | DISCUSSION

Our study shows that translation of iVATS into clinical practice has been successfully initiated at our institution using a safe step-wise process, combining intraoperative C-arm CT scanning and thoracoscopic surgery in a clinical hybrid OR. The main strength of the study was the ability to train other surgeons and professionals, thus leading to successful dissemination of a research technique to clinical care, and ease of use of the procedure without addition of any additional time to the procedure and making a technique for clinical care.

In comparison to our phase I and II clinical trial,^25^ there were no statistical differences in the radiation exposure and time for placement of T-Bars, induction to incision and, incision to closure times among both cohorts. We were able to safely transfer the technology from the clinical research hybrid space to the standard clinical hybrid OR and have demonstrated utility and safety in a larger cohort and with multiple surgeons. We were also able to combine iVATs with other localizing dyes such as ICG and methylene blue to target more that one nodule with a single scan. We also were able to image and localize two nodules in two lobes with one scan with an expiratory breath-hold, thereby decreasing the scan area and limiting radiation dose. The approach was deemed very useful in cases found to have extensive pleural adhesions at time of resection. Our approach can be used not only for difficult-to-palpate nodules, but also in cases with adhesions and previously radiated lung to localize nodules. The approach can also be utilized to localize more than one nodule using a single scan.

The procedure offers a theoretical advantage over other similar techniques such as hookwire localization, which is associated with upto 20% dislodgement and requires an additional procedure for placement. Hookwires can also migrate during lung deflation and can lacerate the lung.^26^ Similarly, other techniques using radiotracer labeling,^18^ or injection of dye such as methylene blue,^27^ ICG, and lipiodol,^20,28^ have been associated with dye spillage into the pleural space and diffusion into the lung, which at time of resection can cause ambiguity as to where the lesion is located. In comparison, iVATS is a single procedure and allows placement of T-bars in the position in which the patient is going to have surgery and is performed as a single procedure with single anesthesia. This allows visualization and localization of nodules in the position in which resection is planned. The fiducial T-bar remains in the lung, while the suture extends from the surface of the lung, this can be combined with additional information from CT scan to triangulate the exact location of the nodule and thus allows targeted resection with optimal margins in most cases. In comparison to bronchoscopic placement of fiducials, this approach allows visualization of the nodule in the position surgery is intended and the suture from the fiducial to extend to the surface of the pleura allowing superior localization as opposed to injection of dye adjacent to a lesion via bronchoscope, which may spread or migrate to adjacent tissues.

With the implementation of lung cancer screening, the detection of number of pulmonary nodules, both solid and part solid and ground glass has increased over the last decade.^29^ The ground glass and partially solid nodules are particularly challenging, as imaging characterization, biopsy, and even surgical resection by standard of care procedures can be difficult. This often results in prolonged follow-up till the lesions are deemed resectable.^5,30–32^ In addition, it is difficult to characterize small partially solid nodules based on imaging alone and there continues to be significant interobserver variability in reporting and characterizing ground glass nodules.^31^ Hence it may not be possible to say with certainty if a nodule is invasive or noninvasive based on imaging alone. Therefore, techniques such as iVATS can enable resection of nodules deemed suspicious for malignancy. In fact, in our cohort having 37 partially solid nodules, 29 (78%) were invasive cancers and five (14%) were noninvasive cancers. Hence, in order to achieve a paradigm shift and to achieve improved survivals in lung cancer, techniques that can help precisely localize and allow anatomical resections at an earlier stage of disease are needed. Even though our procedure was performed in a hybrid OR, with the deployment of new portable C-arms in clinical practice that have CT capability, this technique may be used in any OR.

## 5 | STUDY LIMITATIONS

Our findings need to be interpreted in the context of several limitations. First, the study cohort consisted of selected patients with small pulmonary nodules; and the decision to use iVATS was based on the surgeon’s decision. Hence, a more systemic approach to further refine the indications is needed. Second, procedural time can be variable and can be influenced by patient-specific causes such as adhesions and overall stability during surgery. Therefore, using procedure time as an outcome of success may be not be appropriate in all cases. As we continue to expand our cohort, it may be important to study the learning curve for the operators (surgeons, radiologists, and technologists). Third, it is unclear whether the proposed single-stage approach can be more clinically successful or safe compared with the conventional two-step technique and other methods of localization used both in and outside the hybrid OR. Fourth, our approach requires a hybrid OR and generalizability may be limited. This factor may have also affected the recruitment for the approach. Further research with a larger number of patients is necessary to compare this novel technique with previously established localization methods in terms of diagnostic yields, complication rates, and cost-effectiveness.

## 6 | CONCLUSION

Translation of iVATS into clinical practice has been initiated in our hospital using a safe step-wise process, combining intraoperative C-arm CT scanning and thoracoscopic surgery in a hybrid OR. Future studies will explore the effect of learning curve on the procedure related outcomes as well as a cost-effectiveness analysis comparing VATS with iVATS.

## Figures and Tables

**TABLE 1:** General characteristics of the study cohort

Sex (N)	
Male	20 (40%)
Female	30 (60%)
Age	
Median	65 y
Range	62–80 y
Comorbidities	
Pulmonary	
COPD (PFT – FEV1 predicted percentage range)	33–130
Mild	2 (4%)
Moderate	12 (24%)
Severe	4 (8%)
Cardiac	
Non-ischemic cardiomyopathy	32 (64%)
CAD	2 (4%)
Hypertension	21 (42%)
Prior cancer history	
• Squamous cell carcinoma of the skin	2 (4%)
• Bladder cancer	2 (4%)
• Breast cancer	14 (28%)
• Lung cancer	3 (6%)
• Leukemia /Lymphoma	3 (6%)
• Melanoma	8 (16%)
• Ovarian cancer	2 (4%)
• Sarcoma	1 (2%)
Smoking history (N)	
• ≥30 pack-years	1 (2%)
• Quit>15 y ago	1 (25)
• <30 pack-years	27 (68%)
• Current smokers	4 (8%)
• Never smokers	17 (34%)
Family history of cancer	17 (34%)
Nodule Characteristics	
• Nodule size	0.6–2.7 cm
• Distance to the pleura	0–4.2 cm (mean = 1.3 ± 0.38 cm)
• <1 cm	29 (43%)
• 1–2 cm	13 (26%)
• 2 cm	8 (16%)
Nodule location	
• Left lower lobe	8 (16%)
• Left upper lobe	9 (18%)
• Lingula	2 (4%)
• Right middle lobe	1 (2%)
• Right upper lobe	19 (48%)
• Right lower lobe	11 (22)
Technical factors associated with the study	
Number of scans	46 (1 scan) 4 (2 scans)
Induction to incision (median; range)	89 min (13–256 min)
Time for T-bar placement (median; range)	13.5 min (14–29 min)
Incision to closure time (median; range)	107 min (41–302 min)
Radiation exposure (median; range)	6 mSv; range: 2.9–35 mSv
Length of stay (N; range)	3 d, (1–12 d)
Type of surgery	
• Wedge resection	• 48 (89%)
• Lobectomy	• 4 (7%)
• Segmentectomy	• 2 (4%)
• Lymph node dissection	• 45 (90%)

**TABLE 2:** Lesion morphology and histology of resected nodules

	Solid	Part solid	Pure GGO	Number
Adenocarcinoma spectrum lesions				
TAisN0M0	0	3	3	6 (12%)
TMiaN0M0	0	2	2	4 (8%)
T1aN0M0	2	9	0	11 (21%)
T1bN0M0	3	10	0	13 (25%)
T1cN0M0	1	8	0	9 (17%)
T2aN0M0	0	1	0	1 (2%)
Benign	1	3	0	4 (8%)
Metastases	3	0	0	3 (6%)
Squamous cell carcinoma				
T1aN0M0	0	1	0	1 (2%)
Total	10	37	5	52 (100%)

*Note:* One solid and one pure GGO nodules were not resected

Abbreviation: GGO, ground glass opacity

## Data Availability

The data that support the findings of this study are available on request from the corresponding author. The data are not publicly available due to privacy or ethical restrictions.

## Abbreviations:

iVATS: image-guided video-assisted thoracoscopic resection
CT: computed tomography
GGO: ground glass opacity
ICG: indocyanine green
OR: operating room
